# Supplementary material for: The physiological interactome of TCR-like antibody therapeutics in human tissues
Source: Nat Commun. 2024 Apr 16;15:3271. doi: 10.1038/s41467-024-47062-5 (PMC11021511; doi:10.1038/s41467-024-47062-5)
Supplement: Supplementary file 1 — Supplementary Information [file 41467_2024_47062_MOESM1_ESM.pdf]

SUPPLEMENTARY MATERIAL

SUPPLEMENTARY TABLE 1

Scheinberg sequence	*ESK1 binding*	*Nature*	*Protein origin*	*Expression in the liver*
RMFPNAPYL	yes	control	sp|P19544|WT1_HUMAN	Not detected
RMFWNAPYL	yes	control	sp|P19544|WT1_HUMAN, 1 aa substitution	Not detected
RMFGNAPYL	yes	control	sp|P19544|WT1_HUMAN, 1 aa substitution	Not detected
QLQNPSYDK	no	control	no match	
RAFPNAPYV	no	predicted	sp|P19544|WT1_HUMAN, 2 aa substitution	
RLPPDFFGV	yes	predicted	sp|Q96S15|WDR24_HUMAN	Hepatocytes: medium, Cholangiocytes: low
RLFPLASLV	yes	predicted	sp|Q9NY72|SCN3B_HUMAN	Not detected
RVLPNLVAV	yes	predicted	sp|Q8IY22|CMIP_HUMAN	Hepatocytes: low, Cholangiocytes: low
RMFPNAFYL	yes	predicted	sp|P19544|WT1_HUMAN, 1 aa substitution	Not detected
RVMPCLEAV	yes	predicted	sp|Q6ZMD2|SPNS3_HUMAN	Not detected
RLFPLAWTV	yes	predicted	sp|P20827|EFNA1_HUMAN	Not detected
RLLPLPLGV	yes	predicted	sp|Q8NDA2|HMCN2_HUMAN	Not detected
RLLPLLNSV	yes	predicted	sp|Q8IU68|TMC8_HUMAN; sp|Q5TAT6|CODA1_HUMAN;	Hepatocytes: medium
RLLPLLRTV	yes	predicted	sp|P52798|EFNA4_HUMAN	Hepatocytes: Low
RLWPSLASV	yes	predicted	no match	
RVMPSSFFL	yes	predicted	sp|O75663|TIPRL_HUMAN	Hepatocytes: low
RMFFNAPYL	yes	predicted	sp|P19544|WT1_HUMAN, 1 aa substitution	Not detected
RLWPGQRGV	yes	predicted	sp|Q9UQQ1-6|NALDL_HUMAN	Not detected
RVLPAAFLV	yes	predicted	tr|A0A075B7D9.1|TAF15_HUMAN	Hepatocytes: high; Cholangiocytes: medium
RILPLFQGV	yes	predicted	sp|Q9UN76|S6A14_HUMAN	Not detected
RIIPYLIHV	yes	predicted	sp|P56645|PER3_HUMAN	Hepatocytes: high; Cholangiocytes: medium
RLLPALASV	yes	predicted	sp|Q03154|ACY1_HUMAN	Hepatocytes: high; Cholangiocytes: low
KLMGAISFFI	yes	predicted	sp|Q8NFM4|ADCY4_HUMAN	Hepatocytes: low; Cholangiocytes: low
MLNLLVAIIV	no	predicted	sp|Q8IZF0|NALCN_HUMAN	Not detected
RVIMPCNWWV	yes	predicted	sp|Q9H2S6|TNMD_HUMAN	Not detected
RLLGGMALLL	yes	predicted	sp|Q9NX94-2|WBP1L_HUMAN	Not detected
RLMAMVDVLV	yes	predicted	sp|Q8NFP9|NBEA_HUMAN	Not detected
KTDPTVLLFV	no	predicted	sp|Q9H3R1|NDST4_HUMAN	Not detected
RMFSGVGVYL	yes	predicted	sp|Q969Y0|NXPE3_HUMAN	Not detected
ALLTAALWYI	no	predicted	sp|P17813|EGLN_HUMAN	Not detected
YIHFTPNFLL	no	predicted	sp|Q9UQC9|CLCA2_HUMAN; sp|Q9Y6N3|CLCA3_HUMAN	Not detected

SUPPLEMENTARY FIGURE 1

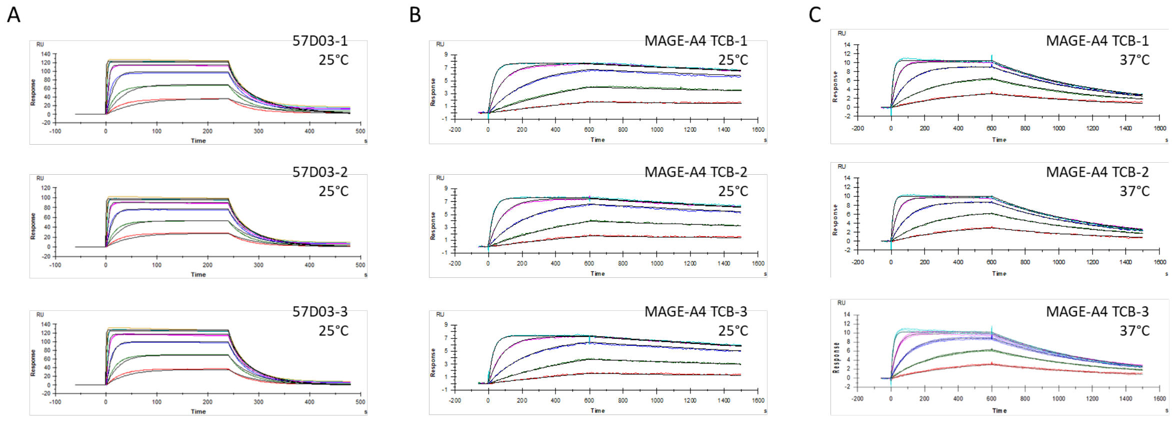

curve	ka (1/Ms)	kd (1/s)	KD (M)
57D03-1	2.86E+06	4.29E-02	1.50E-08
57D03-2	2.56E+06	4.13E-02	1.61E-08
57D03-3	2.38E+06	3.83E-02	1.61E-08
mean	2.60E+06	4.08E-02	1.57E-08
Stdedv	2.42E+05	2.32E-03	6.55E-10

curve	ka (1/Ms)	kd (1/s)	KD (M)
MAGE-A4 TCB-1	5.86E+04	1.69E-04	2.89E-09
MAGE-A4 TCB-2	5.99E+04	2.23E-04	3.73E-09
MAGE-A4 TCB-3	5.83E+04	2.57E-04	4.42E-09
mean	5.89E+04	2.17E-04	3.68E-09
Stdedv	8.62E+02	4.45E-05	7.68E-10

curve	ka (1/Ms)	kd (1/s)	KD (M)
MAGE-A4 TCB-1	1.43E+05	1.60E-03	1.12E-08
MAGE-A4 TCB-2	1.40E+05	1.58E-03	1.13E-08
MAGE-A4 TCB-3	1.45E+05	1.66E-03	1.15E-08
mean	1.43E+05	1.62E-03	1.13E-08
Stdedv	2.35E+03	4.04E-05	1.17E-10

pMHC affinity	CD3 epsilon affinity
K_D = 16 +/- 0.6 nM (25°C)<br>k_a = 2.6 +/- 0.2 E+06 1/Ms (25°C)<br>k_d = 4.1 +/- 0.2 E-02 1/s (25°C)	K_D = 3.7 +/- 0.8 nM (25°C)<br>11.3 +/- 0.1 nM (37°C)<br>k_a = 1.4 +/- 0.0 E+05 1/Ms<br>k_d = 1.6 +/- 0.0 E-03 1/s (25°C)

K_D, equilibrium dissociation constant; k_a, association rate constant ; k_d, dissociation rate constant

Supplementary Figure 1: Affinity profiles for 57D03 and MAGE-A4 TCB (A) Biocore analysis for 57D03 and the MAGE-A4 TCB at indicated temperatures in triplicate analysis. Please see details of the data acquisition can be found in the methods section. (B) Association and dissociation constants for HLA-A*02-GVYDGREHTV (pMHC) and CD3 epsilon.

SUPPLEMENTARY FIGURE 2

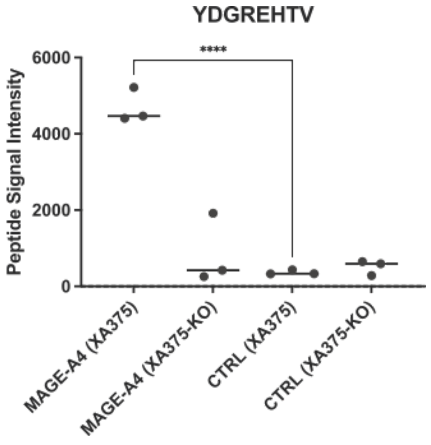

Supplementary Figure 2: Significant enrichment (unpaired t-test, two-tailed $p<0.0001$) of a fragment of the target peptide GVYDGREHTV, the 8mer sequence YDGREHTV, in A375 Xenografts using the MAGE A4 antibody as a bait.

SUPPLEMENTARY FIGURE 3

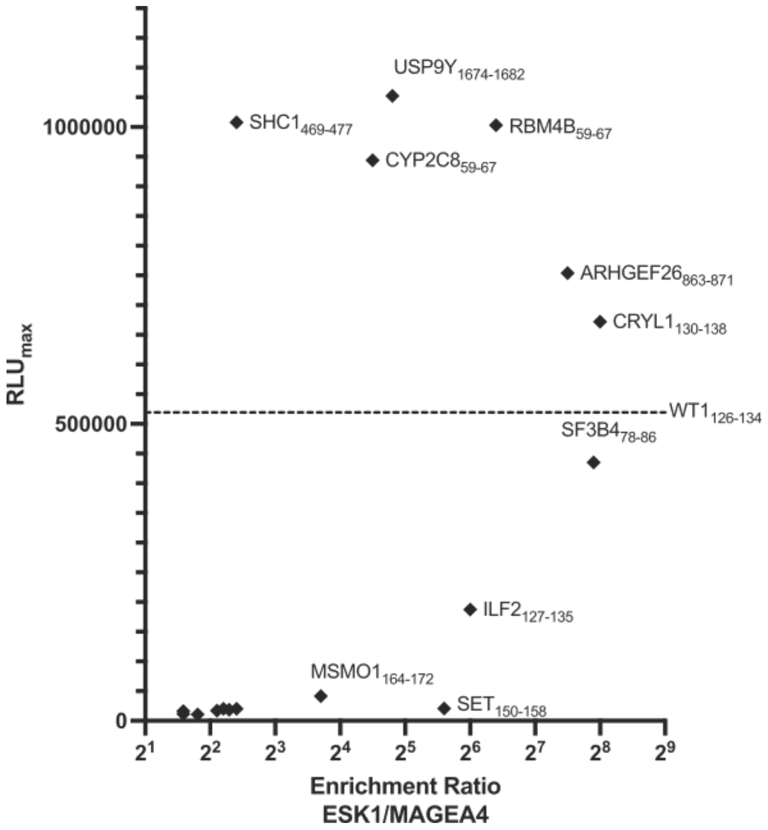

Supplementary Figure 3: No correlation could be observed between the detected ESK1 HLAp enrichment ratio and the measured maximal RLU in a Jurkat reporter assay using peptide-pulsed T2 cells in the presence of ESK1.

SUPPLEMENTARY FIGURE 4

A

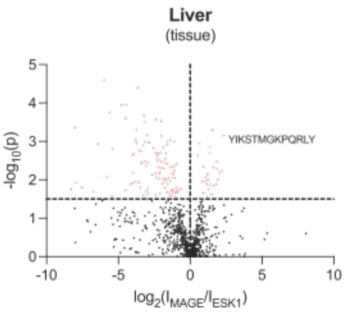

B

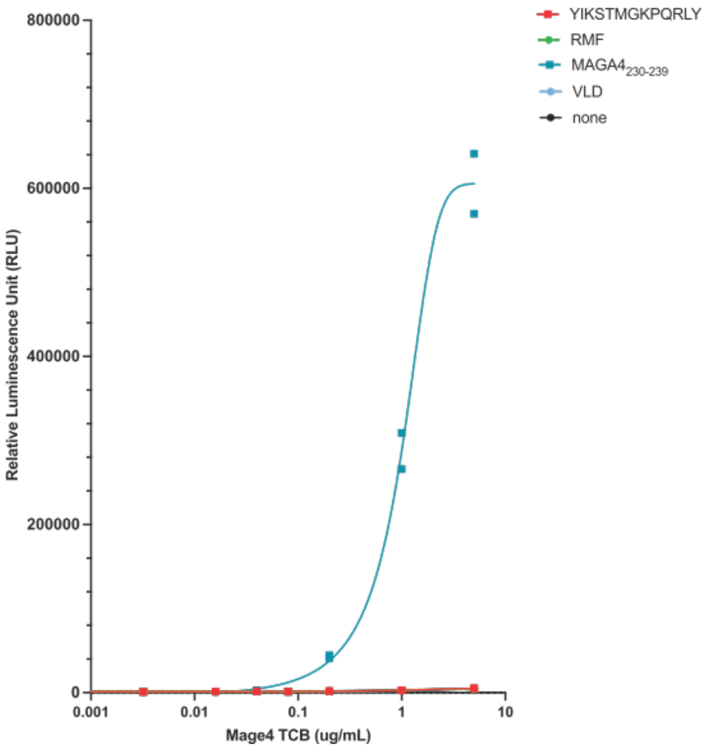

Supplementary Figure 4: Extended data for figure 3. (A) Volcano plot from Figure 3A indicating the most highly significantly enriched peptide sequence (as determined by one-factor ANOVA, -$\log_{10}(p) \geq 1.5$ in triplicate repeats) which does not predict to bind to HLA-A*02:01, YIKSTMGKPQRLY (B) YIKSTMGKPQRLY does not lead to activation of Jurkat NFAT cells in the presence of MAGE-A4 TCB in the NFAT activation assay. Peptides were tested in duplicates, and in parallel to peptides tested in Supplementary Figure 5. $MAGA4_{230-239}$: GVYDGREHTV, positive control peptide; RMF: RMFPNAPYL, VLD: VLDFAPPGA negative control peptides.

SUPPLEMENTARY FIGURE 5

A

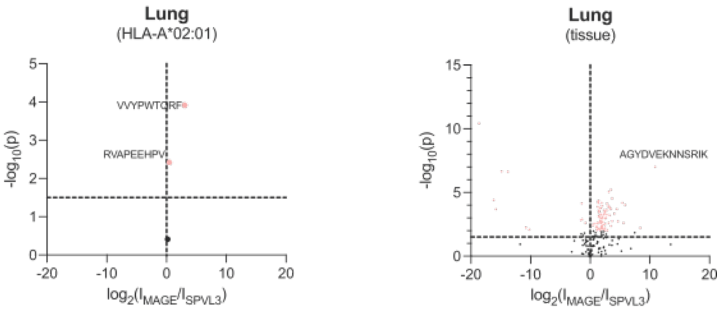

B

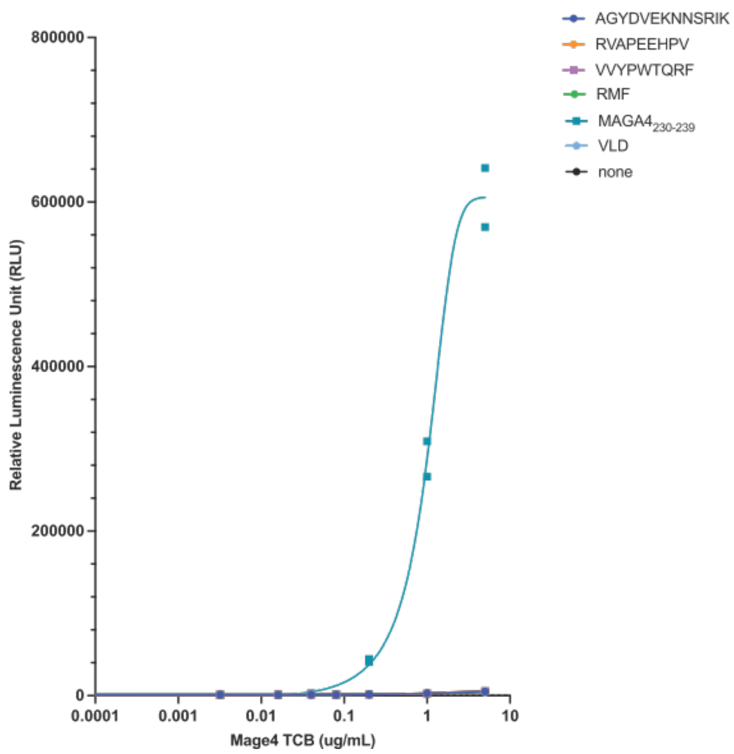

Supplementary Figure 5: Extended data for figure 5. (A) Volcano plot from Figure 5A indicating peptide sequences which are predicted to bind and significantly enriched (one-factor ANOVA, -$\log_{10}(p) \geq 1.5$) in the MAGE-A4 antibody IP (triplicate analysis, left panel, RVAPEEHPV, VVYPWTQRF) and the significantly enriched peptide with highest enrichment factor that does not predict to bind to HLA-A*02:01 (AGYDVEKNNSRIK), right panel. (B) RVAPEEHPV, VVYPWTQRF, and AGYDVEKNNSRIK peptides do not lead to activation of Jurkat NFAT cells in the presence of MAGE-A4 TCB in the NFAT activation assay. Peptides were tested in duplicates, and in parallel to peptide tested in Supplementary Figure 4. $MAGA4_{230\text{-}239}$: GVYDGREHTV, positive control peptide; RMF: RMFPNAPYL, VLD: VLDFAPPGA negative control peptides.
