# Supplementary material for: The physiological interactome of TCR-like antibody therapeutics in human tissues
Source: Nat Commun. 2024 Apr 16;15:3271. doi: 10.1038/s41467-024-47062-5 (PMC11021511; doi:10.1038/s41467-024-47062-5)
Supplement: Supplementary file 3 — Description of Additional Supplementary Files [file 41467_2024_47062_MOESM3_ESM.pdf]

**Description of Additional Supplementary Files**

Title: Supplementary Data 1

Description: LC-MS analysis results for the MAGE-A4 IP experiment in A375 xenograft material (export from Progenesis, v3.0, Waters).

Title: Supplementary Data 2

Description: LC-MS analysis results for the MAGE-A4 IP experiment in liver tissue (export from Progenesis, v3.0, Waters).

Title: Supplementary Data 3

Description LC-MS analysis results for the MAGE-A4 IP experiment in lung tissue (export from Progenesis, v3.0, Waters).

Title: Supplementary Data 4

Description: LC-MS analysis results for the MAGE-A4 IP experiment in colon tissue (export from Progenesis, v3.0, Waters).
