# Supplementary material for: The physiological interactome of TCR-like antibody therapeutics in human tissues
Source: Nat Commun. 2024 Apr 16;15:3271. doi: 10.1038/s41467-024-47062-5 (PMC11021511; doi:10.1038/s41467-024-47062-5)
Supplement: Supplementary file 8 — Reporting Summary [file 41467_2024_47062_MOESM8_ESM.pdf]

nature portfolio

Corresponding author(s): Nicola Ternette
Christian Klein

Last updated by author(s): Mar 8, 2024

Reporting Summary

Nature Portfolio wishes to improve the reproducibility of the work that we publish. This form provides structure for consistency and transparency in reporting. For further information on Nature Portfolio policies, see our Editorial Policies and the Editorial Policy Checklist.

Statistics

For all statistical analyses, confirm that the following items are present in the figure legend, table legend, main text, or Methods section.

n/a	Confirmed	
☐	☒	The exact sample size (*n*) for each experimental group/condition, given as a discrete number and unit of measurement
☐	☒	A statement on whether measurements were taken from distinct samples or whether the same sample was measured repeatedly
☐	☒	The statistical test(s) used AND whether they are one- or two-sided<br>*Only common tests should be described solely by name; describe more complex techniques in the Methods section.*
☒	☐	A description of all covariates tested
☒	☐	A description of any assumptions or corrections, such as tests of normality and adjustment for multiple comparisons
☒	☐	A full description of the statistical parameters including central tendency (e.g. means) or other basic estimates (e.g. regression coefficient) AND variation (e.g. standard deviation) or associated estimates of uncertainty (e.g. confidence intervals)
☐	☒	For null hypothesis testing, the test statistic (e.g. *F*, *t*, *r*) with confidence intervals, effect sizes, degrees of freedom and *P* value noted<br>*Give P values as exact values whenever suitable.*
☒	☐	For Bayesian analysis, information on the choice of priors and Markov chain Monte Carlo settings
☒	☐	For hierarchical and complex designs, identification of the appropriate level for tests and full reporting of outcomes
☒	☐	Estimates of effect sizes (e.g. Cohen's *d*, Pearson's *r*), indicating how they were calculated

Our web collection on statistics for biologists contains articles on many of the points above.

Software and code

Policy information about availability of computer code

Data collection	Mass Spectrometry Data was acquired by Thermo Scientific Tune Software Exactive Series version 2.9.
Data analysis	Structural modelling was performed with the BIOVIA Discovery Studio version 2021. Mass Spectrometry Data was quantitatively analysed using Progenesis QI for Proteomics (Waters), and peptide spectrum sequence assignments were performed using Peaks X (Bioinformatics Solutions). All other data was analysed in Prism 9.3.1. NetMHCpan 4.1 (services.healthtech.dtu.dk) was used to predict HLA peptide sequences to the regarding alleles in each sample. Sequence logos were generated by Seq2logo2.0 and GibbsCluster2.0 (services.healthtech.dtu.dk).

For manuscripts utilizing custom algorithms or software that are central to the research but not yet described in published literature, software must be made available to editors and reviewers. We strongly encourage code deposition in a community repository (e.g. GitHub). See the Nature Portfolio guidelines for submitting code & software for further information.

Data

Policy information about availability of data

All manuscripts must include a data availability statement. This statement should provide the following information, where applicable:

- Accession codes, unique identifiers, or web links for publicly available datasets
- A description of any restrictions on data availability
- For clinical datasets or third party data, please ensure that the statement adheres to our policy

The mass spectrometry proteomics data have been deposited to the ProteomeXchange Consortium via the PRIDE partner repository with the following identifiers: MAGE-A4 TCR-like antibody immunoprecipitation in A375 and A375 MAGE-A4 KO xenograft tissue data are available via ProteomeXchange with identifier

PXD048298 (DOI: 10.6019/PXD048298). MAGE-A4 and ESK1 TCR-like antibody immunoprecipitation data in liver samples are available with identifier PXD048294 (DOI: 10.6019/PXD048294). MAGE-A4 antibody immunoprecipitations in lung and colon tissue are available with identifier PXD048295 (DOI: 10.6019/PXD048295). Source data are provided with this paper.

Human research participants

Policy information about studies involving human research participants and Sex and Gender in Research.

Reporting on sex and gender	This information has not been collected.
Population characteristics	This information has not been collected.
Recruitment	This study did not recruit human participants
Ethics oversight	Human liver samples were obtained from Cytes Biotechnologies (Spain). Human lung and colon samples were obtained from HTCR (Germany). Ethical approval was granted by the Ethics Commission of the Faculty of Medicine in the LMU (number 025-12) and the Bavarian State Medical Association (number 11142), and the Ethical Committee of Hospital Universitari Mútua de Terrassa (number P22 002). Human PBMC were purchased from Lonza (Switzerland), and US collection of samples and commercial distribution is approved and reviewed on an annual basis by the Institutional Review Board, US, under the title: “Prospective collection of non-mobilized leukocytes via leukapheresis for research”.

Note that full information on the approval of the study protocol must also be provided in the manuscript.

Field-specific reporting

Please select the one below that is the best fit for your research. If you are not sure, read the appropriate sections before making your selection.

☒ Life sciences ☐ Behavioural & social sciences ☐ Ecological, evolutionary & environmental sciences

For a reference copy of the document with all sections, see nature.com/documents/nr-reporting-summary-flat.pdf

Life sciences study design

All studies must disclose on these points even when the disclosure is negative.

Sample size	We selected the maximum feasible number of replicates for our experiments. For MS experiments we limited replicate analysis to 3 samples due to the high amount of antibody needed to perform experiments. Killing assays were performed in two independent experiments with two independent human donors in triplicates or quadruplicates (liver).
Data exclusions	No data was excluded.
Replication	Our replication confirmed the results obtained and reported in this study. For qualitative MS data in which peptides are stochastically selected for sequencing, variability of sequences identified in each experiment was observed. We obtained highly reproducible results when only including peptides that were identified in two out of three replicate runs. All experiments were performed in triplicates.
Randomization	In our experiments, randomization did not apply. In contrast, samples were acquired on the LC-MS in sample groups, in order to minimize carryover, but were acquired in batches to minimize technical variation.
Blinding	We did not consider blinding necessary for this study.

Reporting for specific materials, systems and methods

We require information from authors about some types of materials, experimental systems and methods used in many studies. Here, indicate whether each material, system or method listed is relevant to your study. If you are not sure if a list item applies to your research, read the appropriate section before selecting a response.

Materials & experimental systems

n/a	Involved in the study	
☐	☒	Antibodies
☐	☒	Eukaryotic cell lines
☒	☐	Palaeontology and archaeology
☒	☐	Animals and other organisms
☒	☐	Clinical data
☒	☐	Dual use research of concern

Methods

n/a	Involved in the study	
☒	☐	ChIP-seq
☒	☐	Flow cytometry
☒	☐	MRI-based neuroimaging

Antibodies

Antibodies used	ESK1, MAGE-A4 both produced by Roche Pharma and not commercially available. Anti HLA-DQ (SPVL3) was a gift from Anthony Purcell at Monash University.
Validation	Target specificity of the MAGE-A4 antibody which is proprietary to Roche Pharma, has been validated as presented in the preseented study. ESK1 antibody has been developed and validated by Dao et al. 2013, 10.1126/scitranslmed.3005661. HLA-DQ was tested in house for pan-DQ specificity using LABScreen™ (One Lambda).

Eukaryotic cell lines

Policy information about cell lines and Sex and Gender in Research

Cell line source(s)	T2 cell line (source: DSMZ# ACC598); Jurkat NFAT cell line (source: Signosis); A375 (ECACC# 88113005)
Authentication	Cell lines were not authenticated
Mycoplasma contamination	Cell lines are regularly tested for mycoplasma, and confirmed negative.
Commonly misidentified lines (See ICLAC register)	none identified
